# Compound A influences gene regulation of the Dexamethasone-activated glucocorticoid receptor by alternative cofactor recruitment

**DOI:** 10.1038/s41598-017-07941-y

**Published:** 2017-08-14

**Authors:** S. J. Desmet, N. Bougarne, L. Van Moortel, L. De Cauwer, J. Thommis, M. Vuylsteke, D. Ratman, R. Houtman, J. Tavernier, K. De Bosscher

**Affiliations:** 1 Receptor Research Laboratories, Nuclear Receptor Lab, Ghent University, Ghent, Belgium; 2 Receptor Research Laboratories, Cytokine Receptor Lab, Medical Biotechnology Center, VIB, 9000, Ghent, Belgium; 3 Department of Biochemistry, Ghent University, Ghent, Belgium; 4 GNOMIXX bvba, Statistics for Genomics, Ghent, Belgium; 5 PamGene International B.V., ‘s Hertogenbosch, The Netherlands; 6 Roche Global IT Solutions, Roche Polska Sp. z o.o., Warsaw, Poland

## Abstract

The glucocorticoid receptor (GR) is a transcription factor of which the underlying gene regulatory mechanisms are complex and incompletely understood. The non-steroidal anti-inflammatory Compound A (CpdA), a selective GR modulating compound in various cell models, has been shown to favour GR-mediated gene repression but not GR-mediated gene activation. Shifting balances towards only a particular subset of GR gene regulatory events may be of benefit in the treatment of inflammatory diseases. We present evidence to support that the combination of CpdA with Dexamethasone (DEX), a classic steroidal GR ligand, can shape GR function towards a unique gene regulatory profile in a cell type-dependent manner. The molecular basis hereof is a changed GR phosphorylation status concomitant with a change in the GR cofactor recruitment profile. We subsequently identified and confirmed the orphan nuclear receptor SHP as a coregulator that is specifically enriched at GR when CpdA and DEX are combined. Combining CpdA with DEX not only leads to stronger suppression of pro-inflammatory gene expression, but also enhanced anti-inflammatory GR target gene expression in epithelial cells, making ligand combination strategies in future a potentially attractive alternative manner of skewing and fine-tuning GR effects towards an improved therapeutic benefit.

## Introduction

Glucocorticoids (GCs) are steroid hormones derived from cholesterol and secreted by the adrenal glands. As they play a pivotal role in various physiological processes, including glucose metabolism and stress responses, their synthesis is under strict control by the hypothalamic-pituitary-adrenal (HPA) axis and furthermore subjected to circadian and ultradian rhythms. GCs exert their pleiotropic actions through the glucocorticoid receptor α (GR), a ligand-dependent transcription factor (TF) belonging to the nuclear receptor superfamily^1–3^. Due to their hydrophobic nature, GCs diffuse freely through the cellular membrane and bind to the receptor, which in the unliganded state resides in cytoplasm via binding to its chaperone complex. Binding of the receptor to its GC ligand induces a conformational change, followed by nuclear translocation. Once in the nucleus, the GR can activate its target genes by homodimer binding onto cognate DNA sequences, termed glucocorticoid response elements (GRE), a mechanism referred to as transactivation. Additionally, the GR can interfere with the activities of various pro-inflammatory TFs, including nuclear factor κB (NF-kB), without the necessity of DNA binding^4^. This mechanism, called transrepression, is associated with the well-known anti-inflammatory actions of GR. Several coregulators, which can be both coactivators and/or corepressors, are recruited by the receptor to enable GR-mediated transcriptional activities. These proteins have diverse functions, including bridging GR with the transcription initiation complex^5^. Peroxisome proliferator-activated receptor coactivator 1α (PGC-1α) for example, which binds to several members of the NR superfamily, typically enhances the NR-mediated transcription^6^. In contrast, small heterodimer partner (SHP) acts as a transcriptional (co-)repressor of gene expression in a wide array of biological pathways. SHP belongs to the NR0B subfamily, which lack a DNA-binding domain (DBD). This orphan nuclear receptor functions by virtue of its ability to bind directly to multiple NRs and TFs^7, 8^.

GCs, also referred to as corticosteroids, are widely used in the clinic for their anti-inflammatory and immune modulatory actions. Unfortunately, the therapeutic benefits are overshadowed by the occurrence of deleterious side effects and GC resistance. For these reasons, a whole range of so-called selective GR agonists and modulators (SEGRAMs) have been characterized and/or developed, aimed at displaying a more dissociative profile, i.e. favouring GR-mediated transrepression over transactivation. Among those, Compound A (CpdA), a plant-derived compound supporting GR-mediated anti-inflammatory activities, does not stimulate GRE-driven gene expression^9–16^. However, only few dissociative compounds have reached the clinic^17, 18^, and there is still a need for anti-inflammatory therapies with a better therapeutic index.

In the current work, we present data demonstrating an advantageous profile of GR ligand/modulator combinations, more precisely the combination of the full agonist Dexamethasone (DEX) with the SEGRAM CpdA.

## Results

### Combining DEX with CpdA enhances the repression of an NF-κB-dependent reporter and reduces the transactivation activity on a GRE-dependent reporter

**Figure 1:**
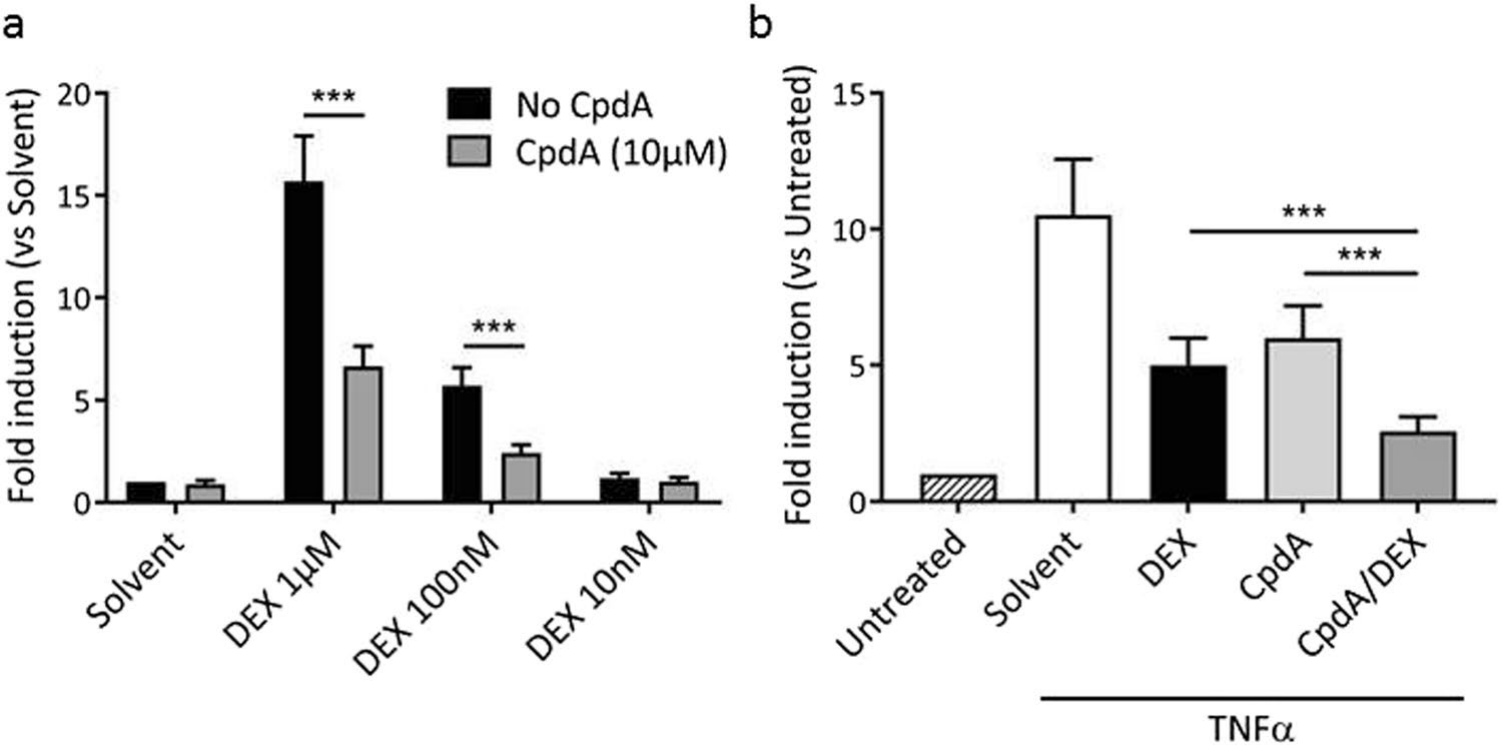
Combining DEX with CpdA enhances the repression of an NF-κB-dependent reporter and reduces the transactivation activity on a GRE-dependent reporter. (**a**) L929sA cells with a stably integrated p(GRE)_2_-50-luc+, a GRE-dependent promoter construct, were pre-incubated with CpdA (10 µM) for 1 h, after which three different concentrations of DEX (10^−6^ M, 10^−7^ M and 10^−8^ M) were added for 5 h. Promoter activities are expressed as relative induction factor versus Solvent/No CpdA. Four independent replicates were performed. Means + SE, obtained as predictions from the HGLMM fitted to the data, are shown on the original scale. (**b**) L929sA cells with a stably integrated p(IL6-κB)_3_-50hu.IL6P-luc+, a NF-κB-dependent promoter construct, were sequentially stimulated with CpdA (10 µM for 6 h), DEX (1 µM for 5 h) and TNFα (2000 units/ml for 4 h) with 1 h between stimulations. Promoter activities are expressed as relative induction factor versus Untreated. Five independent replicates were performed. Means + SE, obtained as predictions from the HGLMM fitted to the data, are shown on the original scale. The significance of ligand combination effects on reporter activity, estimated as differences (on the transformed scale) to the reference level CpdA/DEX, were assessed using a t-test (*p < 0.05; **p < 0.01; ***p < 0.001).

Both the classic agonist DEX and the SEGRAM CpdA inhibit inflammation through interference with the activity of NF-κB^9, 19–21^. In contrast to DEX, however, CpdA does not stimulate GRE-dependent gene expression. This ligand-dependent functional outcome, combined with the finding that CpdA elicits a different GR conformation compared to DEX^9^, led us to postulate that CpdA may modulate a DEX-triggered GR. To test this hypothesis, we investigated the functional outcome upon combining the classic GR ligand, DEX, with the GR modulator, CpdA. In our previous work^9^, where CpdA was added to cells 1 h before DEX, using a GRE-dependent reporter as a read-out, both ligands seemed to functionally compete for the receptor. Oddly, this was not the case when added simultaneously. Hence, we first verified the effect of a pre-treatment with CpdA on the activity of DEX-induced p(GRE)_2_–50-Luc+, a recombinant GRE-driven reporter gene stably integrated in L929sA cells. DEX, in contrast to CpdA, strongly activated the promoter in a dose-dependent manner (Fig. 1a). In line with our earlier findings^9^, CpdA significantly lowers the DEX-induced promoter activity. This result was confirmed in A549 cells, upon using the highest DEX concentration of 1 µM (Supplementary Figure S1a), with no effects on cell viability (Supplementary Figure S1b).

To determine the combined effect of CpdA and DEX on the transrepression potential of GR, we used p(IL6-κB)_3_–50hu.IL6P-luc+, an NF-κB-dependent promoter construct stably integrated in L929sA cells. As expected, both DEX and CpdA efficiently downregulated TNFα-activated reporter activity when added separately (Fig. 1b). Addition of CpdA before DEX however, clearly enhanced this suppression.

### Combining CpdA with a non-saturating DEX concentration suppresses particular inflammatory genes, both at the mRNA and protein level

**Figure 2:**
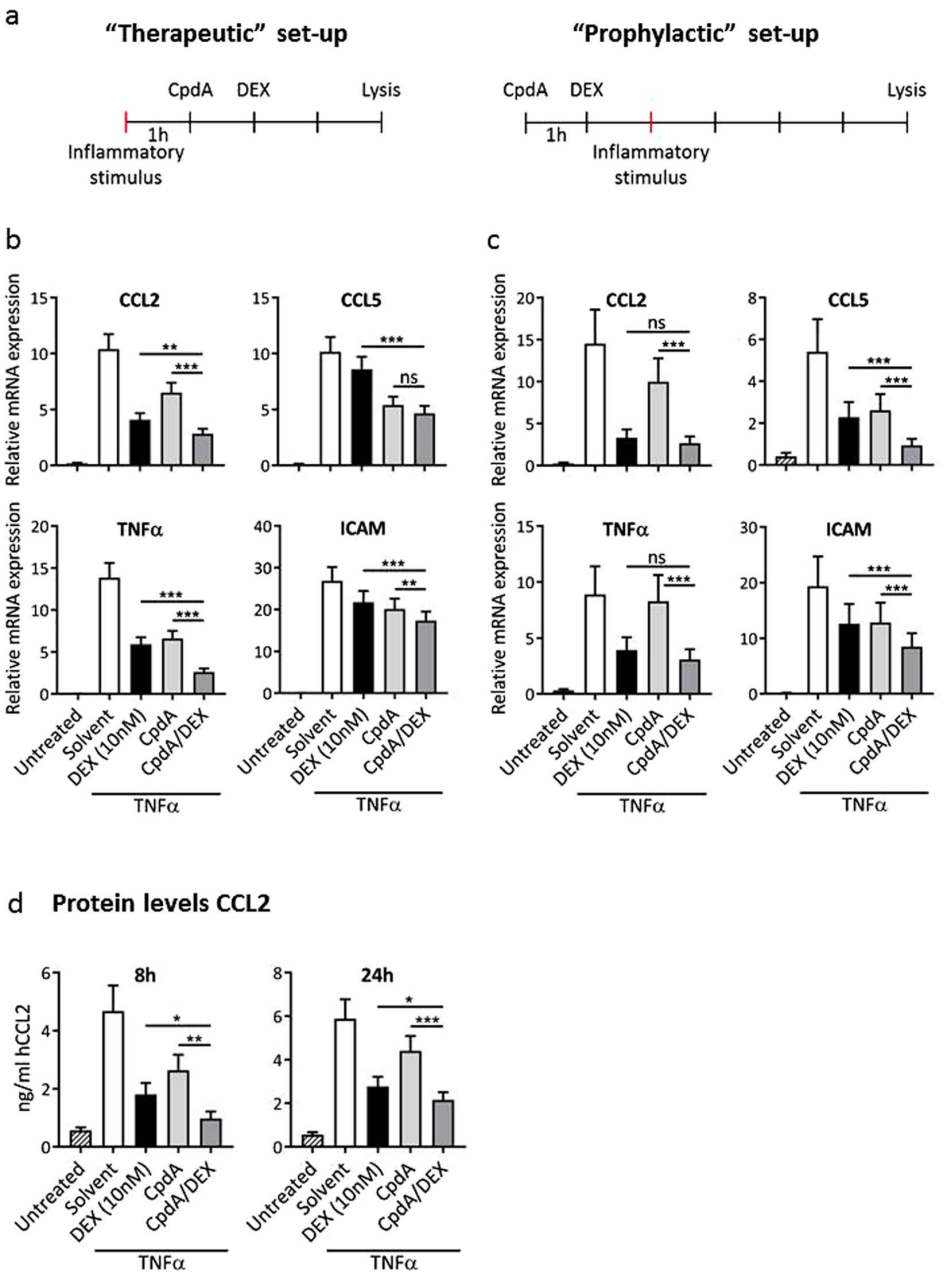
Combining CpdA with a non-saturating DEX concentration suppresses particular inflammatory genes, both at the mRNA and protein level. (**a**) Scheme of the experimental set-ups. (**b**) A549 cells were sequentially stimulated with TNFα (2000 units/ml for 4 h), CpdA (10 µM) and DEX (10 nM) with 1 h between stimulations. Total RNA was extracted and subjected to RT-qPCR. Expression values were normalized to the reference genes *Cyclo* and *HPRT* using qBase+. Five independent replicates were performed. Means + SE, obtained as predictions from the HGLMM fitted to the data, are shown on the original scale for CCL2, CCL5, TNFα and ICAM. (**c**) A549 cells were sequentially stimulated with CpdA (10 µM, for 6 h), DEX (10 nM) and TNFα (2000 units/ml) with 1 h between stimulations. Total RNA was extracted and subjected to RT-qPCR. Expression values were normalized to the reference genes *Cyclo* and *HPRT* using qBase+. At least four independent replicates were performed. Means + SE, obtained as predictions from the HGLMM fitted to the data, are shown on the original scale for CCL2, CCL5, TNFα and ICAM. (**d**) A549 cells were sequentially stimulated with TNFα (2000 units/ml for 8 or 24 h), CpdA (10 µM) and DEX (10 nM) with 1 h between stimulations. Culture supernatant was harvested and measured with sandwich ELISA for CCL2 protein. Three independent replicates, normalized using a standard curve, were performed. Means + SE, obtained as predictions from the HGLMM fitted to the data, are shown on the original scale. The significance of (gene-specific) ligand combination effects on inflammatory gene or protein expression, estimated as differences (on the transformed scale) to the (gene-specific) reference level CpdA/DEX, were assessed using a t-test (*p < 0.05; **p < 0.01; ***p < 0.001).

In a classic set-up to score anti-inflammatory effects *in vitro*, compounds are often administered before the inflammatory stimulus. However, there are two major drawbacks to this methodology. First, this set-up does not answer whether the compound can modulate an ongoing inflammatory response, which is the most likely situation in clinic. Second, it becomes increasingly clear that the sequence in which GC treatment and immune challenge follow each other impacts the final inflammatory outcome. GCs administered prior to the immune challenge could even potentiate the pro-inflammatory response, whereas addition of the GCs after the challenge mostly suppresses ongoing inflammation (reviewed recently in ref. 22). For these reasons, we compared a classic “prophylactic” set-up to a “therapeutic” set-up. The duration (4 h) of the inflammatory challenge was kept identical (depicted in Fig. 2a) to further investigate the combined action of (pre-treated) CpdA and DEX on endogenous gene expression. We further decided to lower the DEX concentration to 10 nM to avoid reaching a maximal response already upon using DEX alone. From a therapeutic perspective, a combination therapy may offer the additional advantage that when the GC dose can be lowered without sacrificing therapeutic effectiveness, the side effects associated with prolonged high-dose GC use can be diminished.

Because airway epithelial cells are at the front line of human defence mechanisms and capable of initiating an innate inflammatory response, we examined the combined effect of DEX (10 nM) and CpdA on TNFα-induced expression in the human respiratory epithelial cell line A549. In line with the data presented in Fig. 1b, combining CpdA with DEX resulted in an enhanced suppression of the pro-inflammatory genes CCL2, CCL5, TNFα and ICAM, compared to DEX or CpdA alone (Fig. 2b and c). Both experimental set-ups displayed a similar overall expression pattern and consistent enhanced suppression when combining (pre-treated) CpdA with DEX. Mild variations between the two set-ups possibly reflect gene-specific sensitivities and/or the fact that the ligands have been on the cells for a longer period of time in the prophylactic set-up, potentially allowing for additional secondary effects. We further measured CCL2 protein using the more physiologically relevant therapeutic set-up, hereby confirming that stronger gene suppression at the mRNA level translates to diminished protein levels. Indeed, after both 8 h and 24 h induction (Fig. 2d), the combined action of (pre-treated) CpdA and DEX resulted in a stronger suppression of CCL2 production, compared to DEX or CpdA stimulation alone.

Finally, we checked whether pre-treatment of CpdA is a prerequisite for these effects, by comparing the results to a set-up in which CpdA and DEX are added simultaneously (Supplementary Figure S2). In both experimental set-ups (depicted in Supplementary Figure S2a), a simultaneous combination of CpdA and DEX could also enhance the suppression of the pro-inflammatory genes CCL2, CCL5, TNFα and ICAM, compared to DEX or CpdA alone (Supplementary Figure S2b,c).

In conclusion, the results with endogenous GR target genes correspond with the above observed enhanced effect on the GR-mediated transrepression of NF-κB-dependent promoter activity, when combining the nonsteroidal GR modulator, CpdA, with the steroidal ligand, DEX.

### The effect of CpdA on DEX-induced gene expression is gene-specific

**Figure 3:**
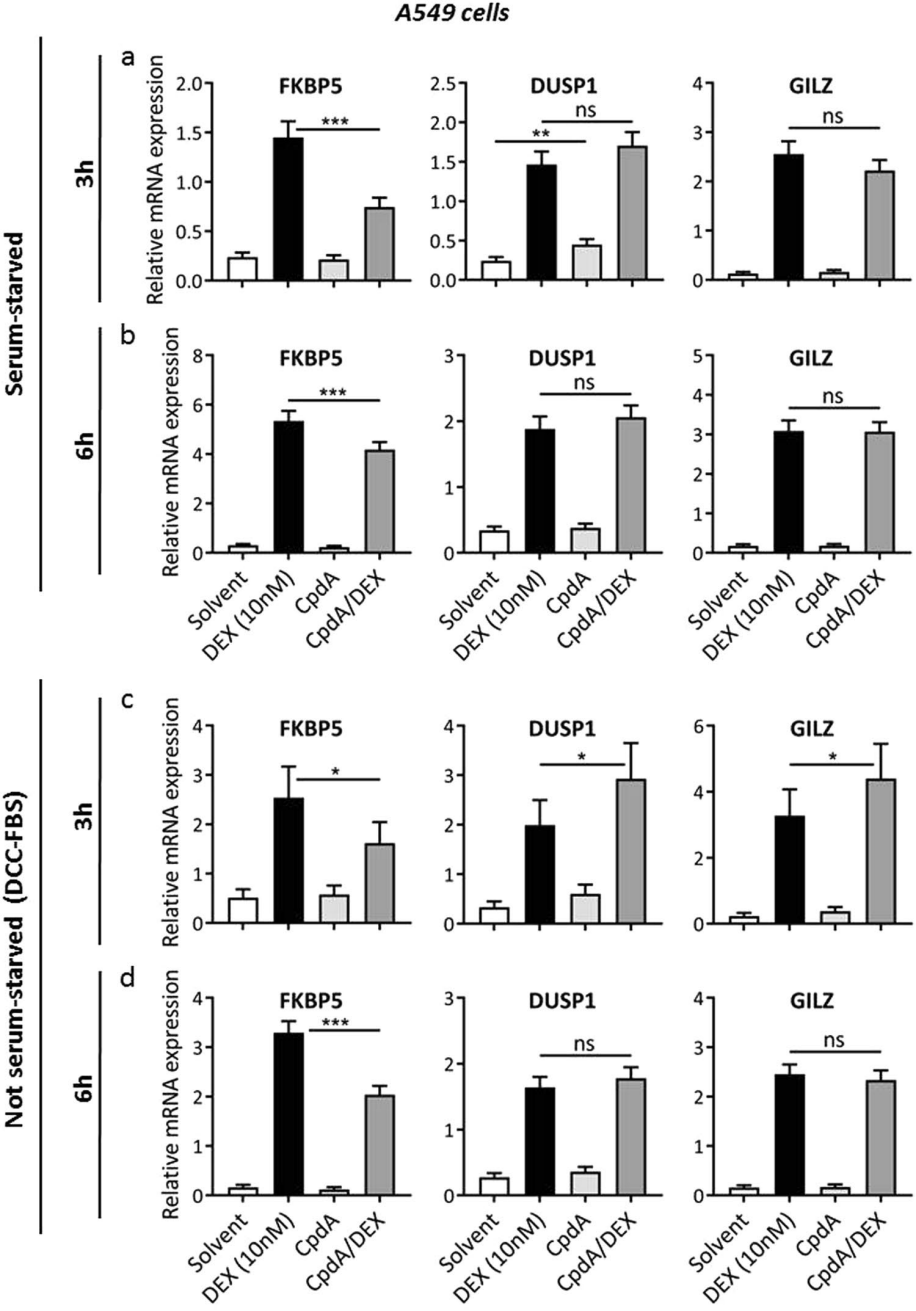
The effect of CpdA stimulation on DEX-induced gene expression is gene-specific. (**a**) Serum-starved A549 cells were pre-incubated with CpdA (10 µM) for 1 h, after which DEX (10 nM) was added for 2 h. Total RNA was extracted and subjected to RT-qPCR. Expression values were normalized to the reference genes *B2M* and *HPRT* using qBase+. At least four independent replicates were performed. Means +SE, obtained as predictions from the HGLMM fitted to the data, are shown on the original scale for FKBP5, DUSP1 and GILZ. (**b**) Serum-starved A549 cells were pre-incubated with CpdA (10 µM) for 1 h, after which DEX (10 nM) was added for 5 h. Total RNA was extracted and subjected to RT-qPCR. Expression values were normalized to the reference genes *B2M* and *HPRT* using qBase+. At least three independent replicates were performed. Means +SE, obtained as predictions from the HGLMM fitted to the data, are shown on the original scale for FKBP5, DUSP1 and GILZ. (**c**) A549 cells were pre-incubated with CpdA (10 µM) for 1 h, after which DEX (10 nM) was added for 2 h. Total RNA was extracted and subjected to RT-qPCR. Expression values were normalized to the reference genes *Cyclo* and *HPRT* using qBase+. Three independent replicates were performed. Means + SE, obtained as predictions from the HGLMM fitted to the data, are shown on the original scale for FKBP5, DUSP1 and GILZ. (**d**) A549 cells were pre-incubated with CpdA (10 µM) for 1 h, after which DEX (10 nM) was added for 5 h. Total RNA was extracted and subjected to RT-qPCR. Expression values were normalized to the reference genes *B2M* and *HPRT* using qBase+. Three independent replicates were performed. Means + SE, obtained as predictions from the HGLMM fitted to the data, are shown on the original scale for FKBP5, DUSP1 and GILZ. The significance of gene-specific CpdA effects on DEX-induced gene expression, estimated as differences (on the transformed scale) to the gene-specific reference level CpdA/DEX, were assessed using a t-test. Furthermore, a t-test was used to test the difference (on the transformed scale) between Solvent and CpdA stimulation (*p < 0.05; **p < 0.01; ***p < 0.001).

Because we observed a markedly diminished transactivation potential of DEX-activated GR upon adding CpdA (Fig. 1a and Supplementary Figure S1a), we wanted to explore this further at the level of endogenous GR target genes. First, we used the non-saturating DEX concentration (10 nM) in both the absence and presence of serum, while applying two time periods (3 h and 6 h of CpdA stimulation) (Fig. 3a–d). As expected, all GRE-dependent genes tested were strongly induced by DEX. In line with the reporter assays (Fig. 1a), CpdA pre-treatment strongly suppressed the DEX-mediated induction of FK506-binding protein 5 (FKBP5), a well-known GRE-dependent gene. Intriguingly, the combined effect appears gene-specific, as the ligand combination either had no effect or even enhanced the expression of other GRE-dependent genes, exemplified by the anti-inflammatory dual specificity phosphatase 1 (DUSP1) and glucocorticoid-induced leucine zipper (GILZ) gene. These trends remain similar across the different starvation methods and time periods. Surprisingly, at the shorter time period in the absence of serum, CpdA alone could induce the expression of DUSP1 (Fig. 3a). At the same time period, the presence of serum enhances the expression level of GILZ when combining CpdA with DEX, albeit not dramatically (Fig. 3, compare panel a and c for GILZ).

Second, we also tested the higher DEX concentration (1 µM) with 6 h of CpdA stimulation, corresponding to the conditions used in the reporter assays of Fig. 1 and Supplementary Figure S1. Supplementary Figure S3a–b shows that at this higher DEX concentration, similar expression patterns and trends are visible upon addition of CpdA. To further investigate the effects on endogenous genes we continued with the non-saturating DEX concentration (10 nM), due to fact that this lower dose corresponds with lower side effects, which is the more desirable situation in the clinic.

Finally, similarly as for the transrepression assays (Supplementary Figure S2), we tested the effect of a simultaneous addition of CpdA and DEX on the endogenous GR target genes in the absence of serum (Supplementary Figure S3c). These results indicate that even when added simultaneously, CpdA can suppress the DEX-mediated induction of FKBP5. Still, the pre-treatment set-up appears to exhibit a more outspoken effect.

In conclusion, these findings indicate that CpdA consistently influences DEX-activated GR-mediated gene expression, under varying cellular conditions and in a gene-specific manner.

### The effect of CpdA on DEX-induced gene expression is more pronounced in mIC_cl2_ cells, in the presence of serum

**Figure 4:**
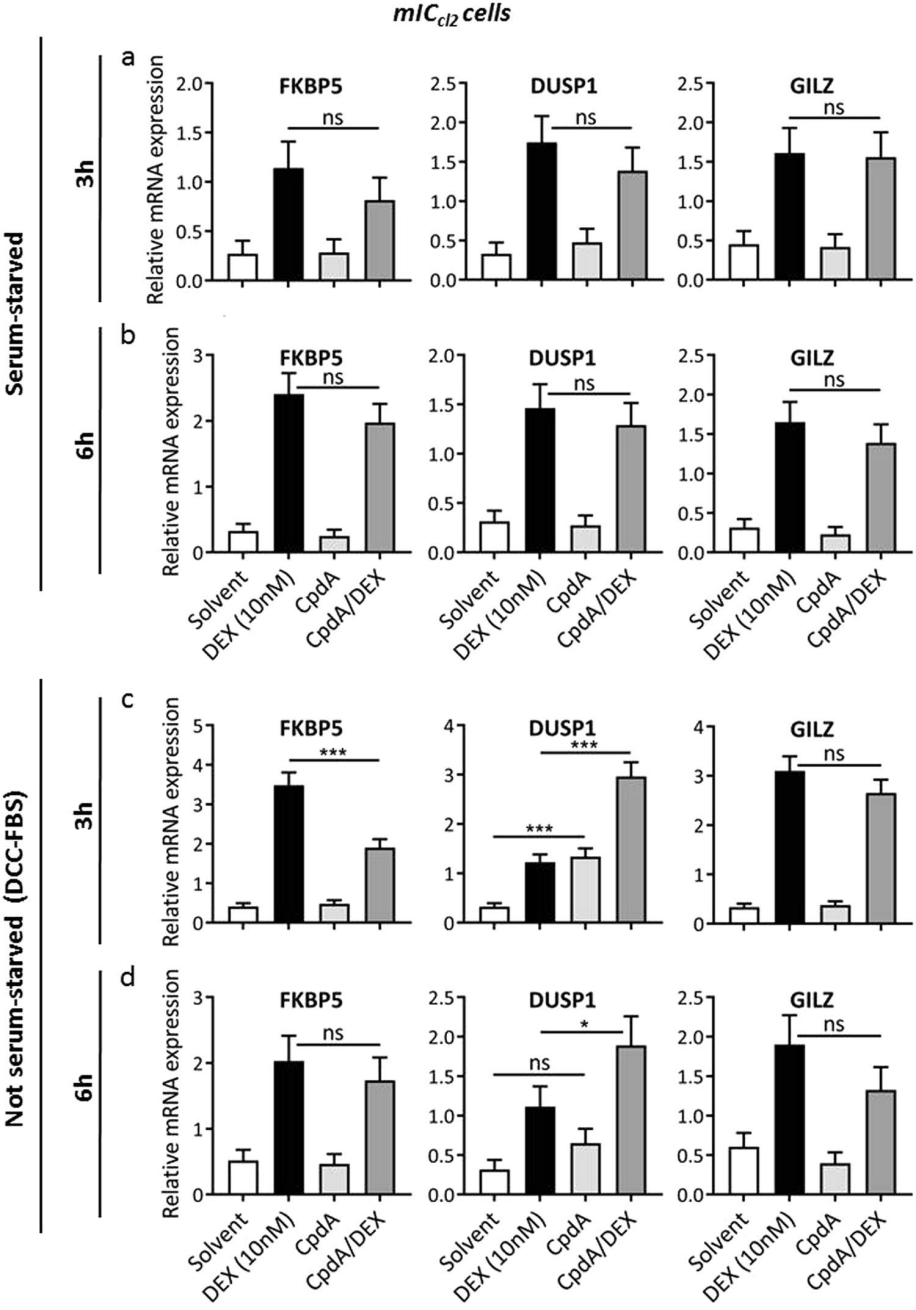
The effect of CpdA on DEX-induced gene expression is more pronounced in mIC_cl2_ cells, in the presence of serum. (**a**) Serum-starved mIC_cl2_ cells were pre-incubated with CpdA (10 µM) for 1 h, after which DEX (10 nM) was added for 2 h. Total RNA was extracted and subjected to RT-qPCR. Expression values were normalized to the reference genes *Cyclo* and *HPRT* using qBase+. Four independent replicates were performed. Means + SE, obtained as predictions from the HGLMM fitted to the data, are shown on the original scale for FKBP5, DUSP1 and GILZ. (**b**) Serum-starved mIC_cl2_ cells were pre-incubated with CpdA (10 µM) for 1 h, after which DEX (10 nM) was added for 5 h. Total RNA was extracted and subjected to RT-qPCR. Expression values were normalized to the reference genes *Cyclo* and *HPRT* using qBase+. Three independent replicates were performed. Means + SE, obtained as predictions from the HGLMM fitted to the data, are shown on the original scale for FKBP5, DUSP1 and GILZ. (**c**) mIC_cl2_ cells were pre-incubated with CpdA (10 µM) for 1 h, after which DEX (10 nM) was added for 2 h. Total RNA was extracted and subjected to RT-qPCR. Expression values were normalized to the reference genes *Cyclo* and *HPRT* using qBase+. Three independent replicates were performed. Means + SE, obtained as predictions from the HGLMM fitted to the data, are shown on the original scale for FKBP5, DUSP1 and GILZ (**d**) mIC_cl2_ cells were pre-incubated with CpdA (10 µM) for 1 h, after which DEX (10 nM) was added for 5 h. Total RNA was extracted and subjected to RT-qPCR. Expression values were normalized to the reference genes *Cyclo* and *HPRT* using qBase+. Three independent replicates were performed. Means + SE, obtained as predictions from the HGLMM fitted to the data, are shown on the original scale for FKBP5, DUSP1 and GILZ. The significance of gene-specific CpdA effects on DEX-induced gene expression, estimated as differences (on the transformed scale) to the gene-specific reference level CpdA/DEX, were assessed using a t-test. Furthermore, a t-test was used to test the difference (on the transformed scale) between Solvent and CpdA stimulation (*p < 0.05; **p < 0.01; ***p < 0.001).

Next, we wondered whether CpdA influenced DEX-induced GR target gene expression in the same manner, using another epithelial cell line. For this purpose, we studied mIC_cl2_ cells, which are positioned as a first line of defence in the small intestinal tissue. Both epithelial cell lines express GR protein levels to a similar extent (see Supplementary Figure S4). In analogy with Fig. 3, we tested both starvation methods and time periods in the mIC_cl2_ cells, and measured mRNA levels of FKBP5, DUSP1 and GILZ. As expected, DEX induced the expression of all three genes. This is in contrast to CpdA, at least for FKBP5 and GILZ (Fig. 4). Surprisingly, in the presence of serum, CpdA alone significantly induced DUSP1 to a similar extent as the non-saturating DEX concentration (Fig. 4c). This gene-specific induction at the shorter time period is even more pronounced than in the A549 cells, in which a similar effect was observed under serum-starved conditions (Fig. 3a). In the absence of serum, all CpdA/DEX combination effects seemed to be blunted in the mIC_cl2_ cells (Fig. 4a,b). However, in the presence of serum, DEX-mediated upregulation of FKBP5 expression was significantly downregulated by CpdA, whereas DUSP1 expression was significantly enhanced (Fig. 4c). These results correspond nicely with the effects in the A549 cells (Fig. 3c). In contrast, the expression of GILZ in the mIC_cl2_ cells was not enhanced by combining CpdA with DEX, compared to DEX alone. At the longer time period (Fig. 4d), also in mIC_cl2_ cells the effects again become less pronounced, in line with the results in Supplementary Figure S3c for FKBP5 in A549 cells.

In conclusion, the effect of CpdA on DEX-induced gene expression is both gene- and cell type-specific.

### CpdA suppresses S211 phosphorylation of DEX-activated GR

Looking further into potential underlying mechanisms that may help explain the differential effects of the ligand combination compared to DEX alone, we speculated on a role for receptor phosphorylation and focused on phosphorylated Serine (Ser) 211 of GR, since this modification is considered a hallmark for GR transactivation^23, 24^.

**Figure 5:**
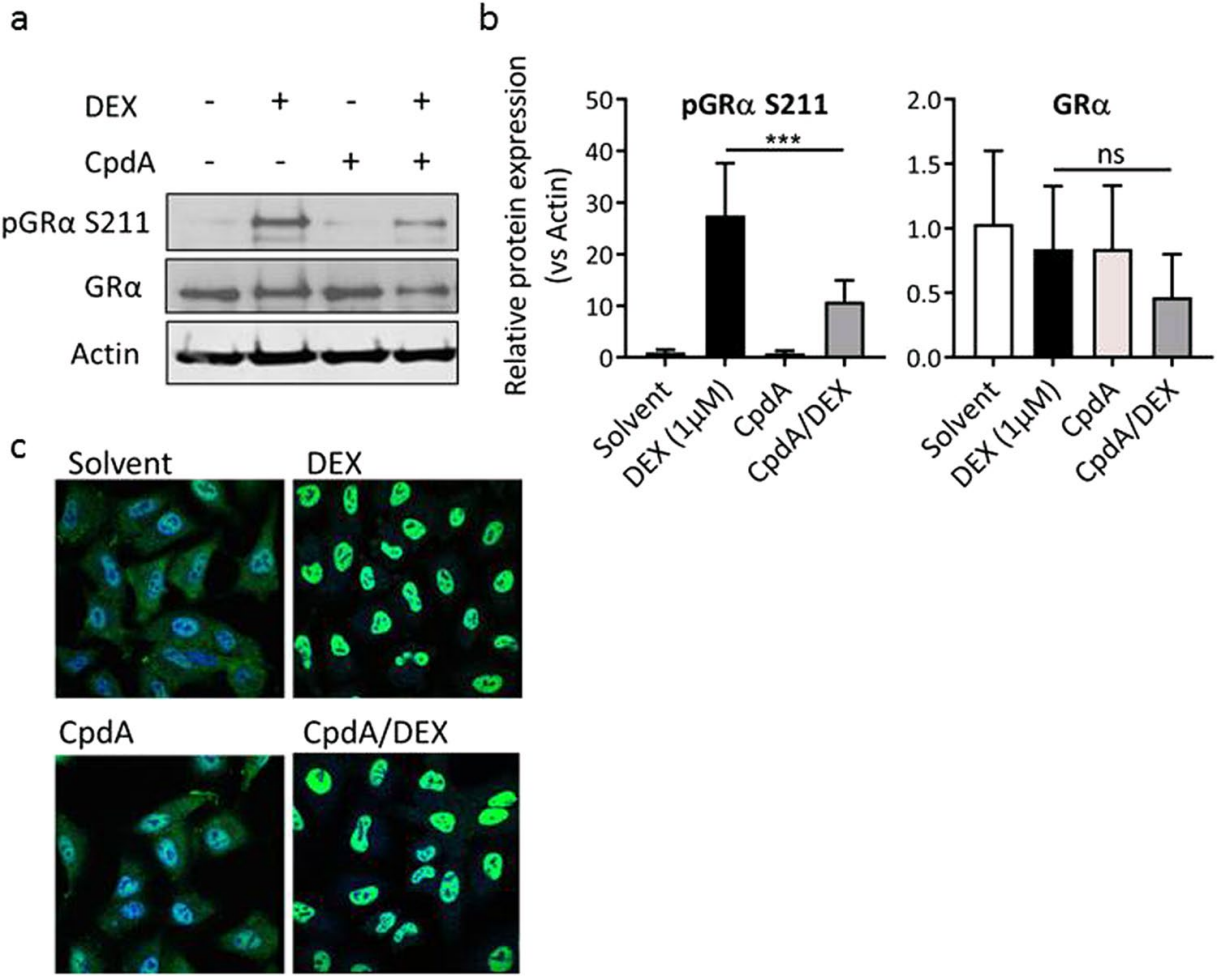
CpdA suppresses S211 phosphorylation of DEX-activated GR, but has no effect on its nuclear translocation. (**a**) A549 cells were pre-incubated with CpdA (10 µM) for 1 h, after which DEX (1 µM) was added for 1 h. Total cell lysates were prepared and subjected to Western Blot analysis. Actin served as a loading control (n = 3, representative figure). (**b**) Quantification of (**a**). Expression values, obtained with Image J analysis of three independent replicates, were normalized to the loading control Actin. Means + SE, obtained as predictions from the HGLMM fitted to the data, are shown on the original scale for pGRα S211 and GRα. (**c**) A549 cells were pre-incubated with CpdA (10 µM) for 1 h, after which DEX (1 µM) was added for 1 h. After fixation, cells were subjected to immunostaining with anti-GR, followed by anti-rabbit-Alexa 488 as a secondary Ab. DAPI staining was used to visualize nuclei (n = 2, representative figure). The significance of CpdA effects on DEX-induced protein expression, estimated as differences (on the transformed scale) to the reference level CpdA/DEX, were assessed using a t-test (*p < 0.05; **p < 0.01; ***p < 0.001).

As expected, activation of the receptor with DEX clearly enhanced the specific phosphorylation of Ser-211 after 1 h of induction, compared to the background level (Fig. 5a). In contrast, CpdA alone did not induce phosphorylation of Ser-211, which correlates with the inability of CpdA alone to support GR-mediated transactivation of most GRE-driven genes (a.o. FKBP5 and GILZ). Yet, not all GRE genes behave similar as CpdA alone was found to induce DUSP1 in a context-dependent manner (Figs 3 and 4). Combining the two ligands resulted in a decreased phosphorylation compared to DEX treatment alone. Equal sample loading was verified using a non-phosphorylation-specific antibody and protein levels of actin served as an additional loading control. Quantification of the Western Blots was performed to ensure the accuracy of the conclusions (Fig. 5b). The signal of the total GR or the phosphorylated GR protein expression was normalised to the expression of the actin loading control. Correspondingly, we observed a significant decrease in Ser-211 GR phosphorylation when combining CpdA and DEX, compared to DEX treatment alone. In contrast, no significant changes were apparent concerning the total GR protein levels.

These results indicate that combining CpdA with DEX diminishes GR phosphorylation at Serine 211, which may result in a context-dependent diminished transactivation potential of the receptor.

### CpdA leaves nuclear translocation of DEX-bound GR unhampered

Using indirect immunofluorescence to detect endogenous GRα in A549 cells, Fig. 5c illustrates that the unliganded receptor mainly resides in the cytoplasm. Following DEX treatment, the receptor clearly translocated to the nucleus. CpdA resulted in a mixed phenotype, with both nuclear and cytoplasmic GR proteins. Addition of CpdA before DEX did not affect the subcellular localisation compared to DEX induction alone. These findings indicate that a reduction in GR phosphorylation at Ser-211, by combining CpdA with DEX, does not impact the ability of the receptor to translocate to the nucleus.

### Combining CpdA with DEX results in a distinct GR cofactor profile

**Figure 6:**
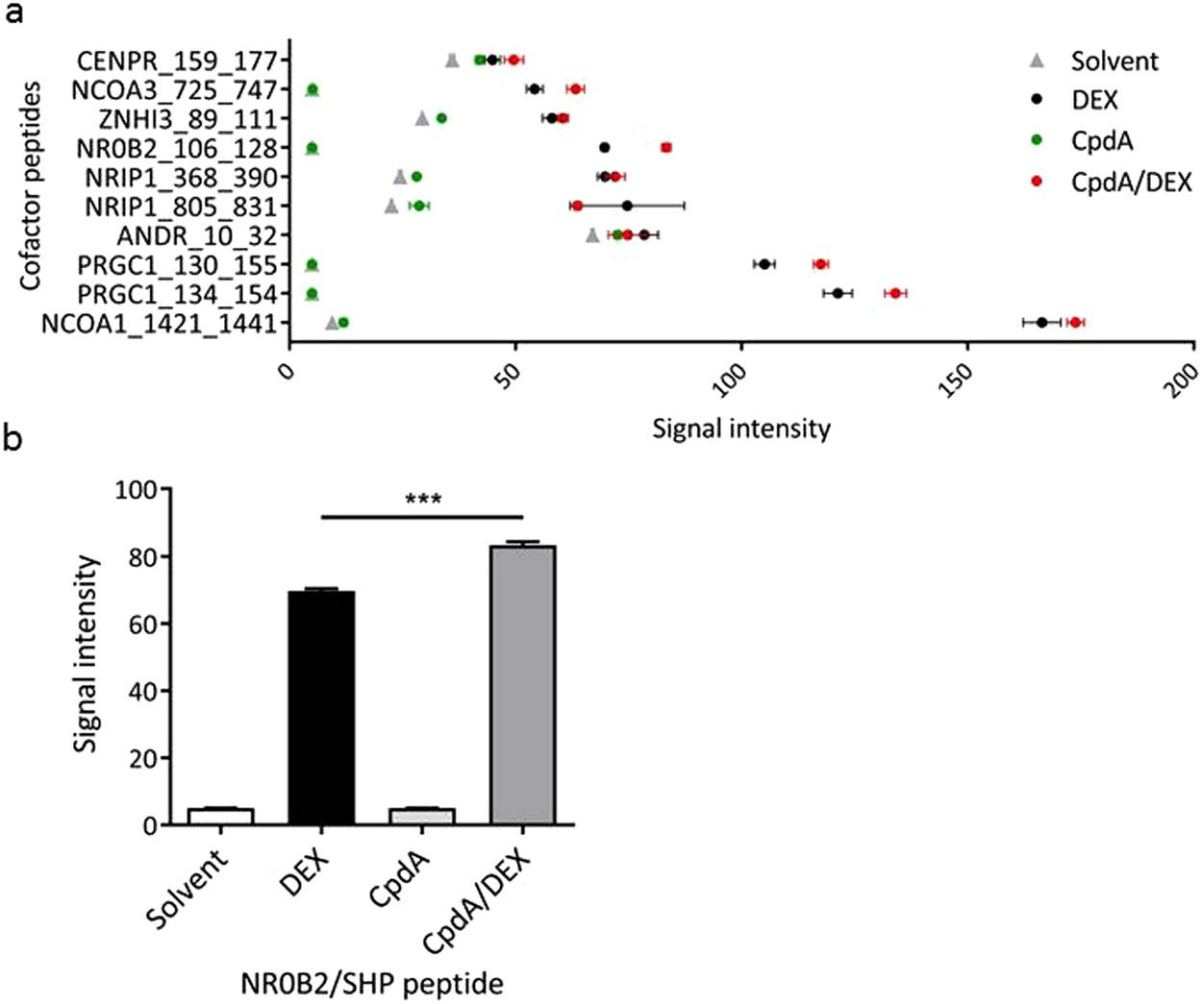
Combining CpdA with DEX results in a distinct GR cofactor recruitment profile. (**a**) MARCoNI assay result with recombinant GRα-LBD-GST stimulated with DEX (1 µM) and/or CpdA (10 µM). Interaction between the recombinant GR and the immobilized cofactor-derived peptides are detected with an anti-GST antibody labeled with Alexa 488, after several washing steps to eliminate unbound GR proteins. The top 10 interacting peptides with DEX-bound GR-LBD are shown here. Data was normalized via LOESS regression (Mean + SEM, n = 3). (**b**) Detail result of (**a**): SHP (=NR0B2) peptide interacts stronger with CpdA/DEX-stimulated GR compared to DEX-bound GR. Statistical analysis via one-way ANOVA (p-value < 0.001) with post-hoc t-test (corrected for multiple testing) (***p < 0.001).

As cofactors regulate the transcriptional activity of nuclear receptors (NR), we asked whether combining CpdA with DEX resulted in a different cofactor recruitment pattern compared to either ligand alone. To this end, we applied the Microarray Assay for Realtime Coregulator-Nuclear receptor Interaction (MARCoNI) technology, in which interactions between a recombinant GRα-LBD-GST and peptides derived from known coactivator or corepressor proteins are monitored^25, 26^. Coregulator-derived peptides are immobilized on a solid support, building a micro-array, and incubated with the recombinant GR and a GST-specific antibody coupled with a fluorophore. Interaction strengths between the receptor and the NR binding motif in the peptides correlate with the amount of immobilized fluorescent label^26^. As expected, DEX alone recruited several coregulators to GR, of which the 10 strongest binders are presented in Fig. 6a. These include mostly coactivators, such as nuclear receptor coactivator 1 (NCoA1) and PGC-1α. CpdA alone surprisingly showed no significant additional classic coregulator recruitment compared to the unliganded receptor profile. However, in line with a differential effect on gene expression (Figs 2–4), CpdA did subtly influence the cofactor profile of a DEX-bound receptor. We selected one peptide of the strong DEX-GR binders, i.e. NR0B2_106_128, which showed a significantly stronger interaction with the DEX/CpdA-activated GR than with the DEX-bound GR (Fig. 6b). This peptide is derived from the protein nuclear receptor subfamily 0 group B member 2 (NR0B2), more frequently referred to as SHP.

### CpdA enhances the interaction of DEX-bound GRα with the cofactor SHP

**Figure 7:**
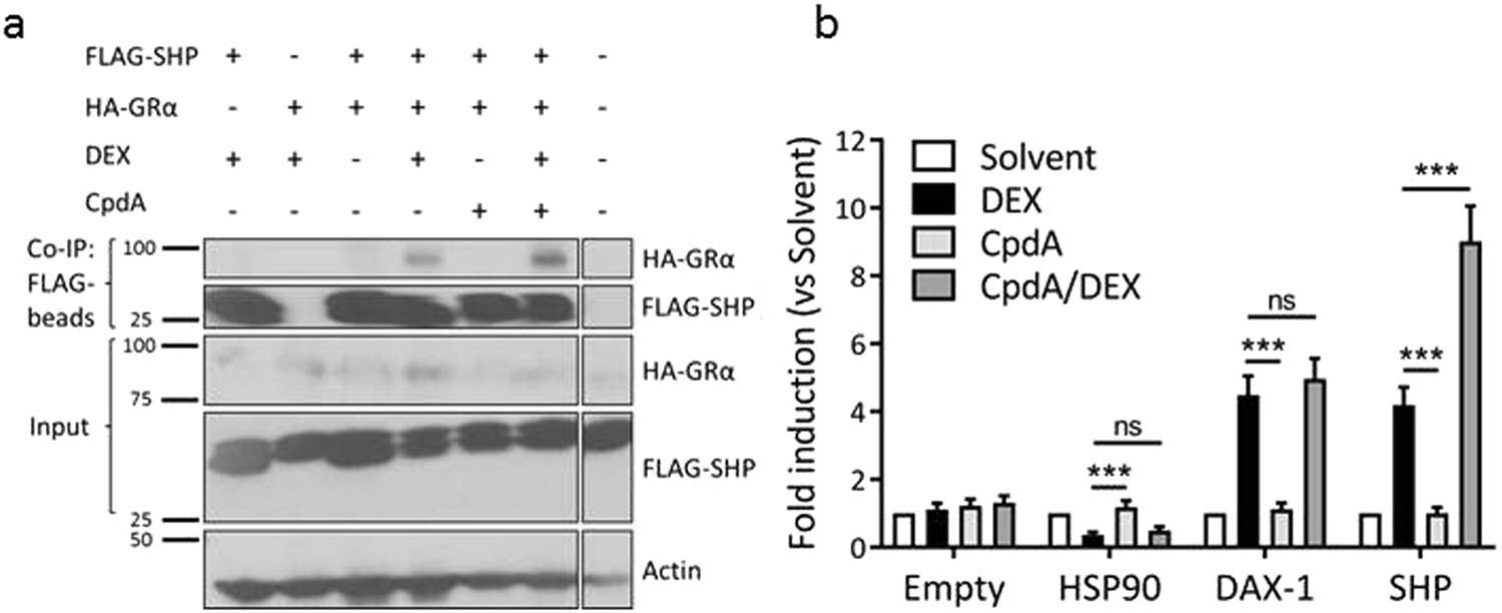
CpdA enhances the interaction of DEX-bound GRα with the cofactor SHP (**a**) Co-immunoprecipitation between Flag-SHP and HA-GRα overexpressed in HEK293T cells (ratio GR/SHP was 1/5). Cells were stimulated with CpdA (10 µM) 1 h before DEX (1 µM) and lysed 3 h after DEX stimulation (n = 4, representative figure). (**b**) Mammalian protein-protein interaction technique (MAPPIT). Empty prey is used as negative control, HSP90 prey as selective control. Cells were stimulated with leptin (100 ng/ml) and leptin in combination with DEX (1 µM) and/or CpdA (10 µM) for 24 h or were left untreated. Luciferase measurements were performed in triplicate (normalized by β-galactosidase expression) and normalized by untreated values. Reporter activities are expressed as relative induction factor versus Solvent. Three independent replicates were performed. Means + SE, obtained as predictions from the HGLMM fitted to the data, are shown on the original scale. The significance of ligand effects on binding activity, estimated as differences (on the transformed scale) to the reference level DEX, were assessed using a t-test (*p < 0.05; **p < 0.01; ***p < 0.001).

To validate the above selected differential binder, we performed co-immunoprecipitation analysis in HEK293T cells with overexpressed Flag-SHP and HA-GRα in the presence or absence of CpdA and/or DEX. The interaction between SHP and GR was, as expected, ligand-dependent (Fig. 7a), and enhanced when combining CpdA with DEX. In addition to co-immunoprecipitation, we applied another protein-protein interaction technique, namely the Mammalian Protein-Protein Interaction Trap (MAPPIT)^27, 28^. MAPPIT is a two-hybrid system based on the restoration of a dysfunctional cytokine receptor signalling pathway through interaction between ‘bait’ and ‘prey’ chimeras, followed by a STAT3-dependent luciferase read-out. The GRα was used as a bait protein together with an empty control prey protein, the chaperone heat shock protein 90 (HSP90) and the protein of interest, SHP. We also included a family member of SHP, DAX-1 (dosage-sensitive sex reversal-adrenal hypoplasia congenita critical region on the X-chromosome, gene 1), to test whether the combined ligand effect is specific to SHP. As expected, upon activation of GR with DEX, the interaction between GR and its chaperone protein HSP90 was lost (Fig. 7b). This was not the case for CpdA, as described previously in Beck *et al*.^29^. However, combining CpdA with DEX disrupted the interaction with HSP90, similar to DEX activation alone. Concerning the interaction with SHP, we confirmed a stronger interaction to the receptor with the ligand combination than with DEX alone. This enhancement was specific to SHP, as the family member DAX-1 did not differ in binding to GR comparing DEX with CpdA/DEX. In conclusion, we show that CpdA enhances the DEX-mediated recruitment of the cofactor, SHP, to GR.

## Discussion

Therapeutic use of classic glucocorticoids, such as Dexamethasone (DEX), is overshadowed by side effects and the occurrence of GC resistance. In the current work, we present data demonstrating an advantageous profile of GR ligand/modulator combinations, more precisely the combination of the full agonist DEX with the SEGRAM Compound A (CpdA).

Anti-inflammatory activities of CpdA treatment alone have already been established in diverse animal disease models^9–15^ and *ex vivo*, in human patient tissue^16^. GR-independent effects of CpdA have been reported^15, 30–32^, yet their underlying mechanisms are to date unresolved and may constitute cell-specific phenomena^30^. Given an observed GR-dependency of the suppression of inflammatory mediators by CpdA in particular cell models^9, 12^, anti-inflammatory effects of CpdA are at least partially GR-dependent.

Since the effects of CpdA and DEX are clearly not superimposable, we set out to study potential unique gene-modulatory properties of a combined effect. We discovered that combining this SEGRAM with DEX was capable to further enhance the NF-κB-targeting transrepression potential of the receptor in L929sA murine fibroblast cells. These results were further validated in A549 cells, showing strong suppression by the combined action of CpdA and a non-saturating DEX concentration (10 nM) on the TNFα-induced gene expression of TNFα, ICAM, CCL5 and CCL2. For the latter gene, we confirmed the combined effect was also visible at the protein level, supporting that this unique gene regulatory profile can be linked to functional chemokine protein levels.

Although CpdA does not stimulate GRE-driven reporter gene expression on its own, it can efficiently downregulate GRE-dependent promoter activity induced by DEX-activated GR, confirming previous results that were suggestive of a competitive effect between DEX and CpdA for binding to GR^9^. Remarkably, the order of ligand addition was important in the reporter assays, as simultaneous addition of CpdA and DEX had no effect compared to DEX alone^9, 33^. However, using the more physiologically relevant read-out of mRNA expression levels, simultaneous addition resulted, at least for the GR targets tested here, in similar combined effects. Recently, CpdA combined with fluocinolone acetonide (FA), a glucocorticoid used mainly for topical application, was tested on GRE-dependent reporter constructs in murine immortalized keratinocytes^34^. In line with our results using DEX, CpdA strongly suppressed the FA-induced GRE-dependent promotor activity, supporting that the competitive effect is not GC ligand-specific. Additionally, a recent report by Malaise and colleagues^35^ studied the potential of ligand combinations using synovial fibroblasts, derived from patients with osteoarthritis (OA). In this study, *ex vivo*, long-term (7 days) treatment with CpdA led to lower prednisolone-induced leptin secretion and leptin receptor expression. The GC-mediated induction of leptin, which plays a detrimental role in OA, is believed to be caused by an enhanced GRE-dependent expression of GILZ, which was not induced by CpdA^36^. Corresponding to our findings at the endogenous gene level, in both aforementioned studies the order of ligand addition did not play a role since effects of CpdA together with FA or prednisolone were observed using simultaneous stimulation. The overall conclusions of these independent studies underscore the hypothesis that CpdA is capable of changing the activity of an agonist-bound GR.

Reporter gene experiments were confirmed at the mRNA level for FKBP5, the 51 kDa member of the immunophilin protein family and a bona fide GRE-driven glucocorticoid target, in both lung and intestinal epithelial cells. However, the influence of CpdA on the transactivation potential of DEX-activated GR appeared to be promoter- and cell type-dependent, as two GRE-dependent anti-inflammatory genes were unaffected or even enhanced. The induction of the GILZ gene upon CpdA/DEX was further only apparent in the A549 cells at certain culture conditions. DUSP1 expression - also known as MAP kinase phosphatase-1 (MKP1) - was significantly enhanced in both epithelial cells, when combining CpdA with DEX. This enhancement was even more pronounced in the mIC_cl2_ cells, compared to the A549 cells. GRE-mediated DUSP1 expression in intestinal tissue was proven crucial to protect against TNFα-induced inflammation^37^. Consequently, our results underscore the potential advantage of selectivity for specific GRE elements following ligand combinations. Fitting previous conclusions that CpdA does not stimulate GRE-driven gene expression^11, 12, 38^, we observed no effect by CpdA alone on the reporter gene assays and particular endogenous genes. Surprisingly however, at the shorter time period and depending on the cellular context, we did observe a mild induction of DUSP1 expression by CpdA alone. This gene-specific induction was even more pronounced in the intestinal epithelial cells, where we observed a strong enhancement of DUSP1 expression by CpdA alone, comparable to levels obtained with a non-saturating concentration of DEX alone. This is in contrast to the lack of DUSP1 induction by CpdA in another intestinal epithelial cell line Caco-2 cells, in which GR was however transiently transfected^39^. This differential effect of CpdA may be due to differences in GR levels, which could result in changes in ligand sensitivity, as proposed by Robertson *et al*.^40^. Recently, another study^31^, using airway smooth muscle cells, reported a CpdA-induced expression of DUSP1, but not GILZ, both at the mRNA and protein level. Similar to our results, this occurred after a short time period, but in a steroid-resistant context, and was found to be GR-independent^31^. As a final explanation, CpdA may degrade more quickly in the cells as compared to the stable steroidal DEX, which is why a consideration of future treatment strategies based on the advantages of a dual GR ligand/modulator combination would definitely require stabler SEGRAM molecules^15^.

Ligand competition for GR molecules in the cell has been suggested as an underlying mechanism for the combined effect, whereby CpdA could act as a partial GR antagonist^9, 34, 35^. In the current work, we present evidence for potential other mechanisms that may help explain the differential effect of the ligand combination compared to DEX alone.

CpdA does not elicit the same phosphorylation status of the GR as DEX does, and it induces a different conformation of GR following limited trypsin protease digestions^9^. We hence hypothesized that CpdA may also modulate a DEX-triggered GR, subtly changing GR conformation and thus leading to functional differences. This hypothesis would fit with the finding of modest differences in cofactor recruitment patterns using the MARCoNI assay comparing a CpdA/DEX-activated GR to DEX activation alone. As this assay was done using recombinant GRα-LBD, any change in cofactor binding is conformation-dependent, as no other cellular factors are present. Furthermore, we found a decrease in Serine-211 phosphorylation after combined treatment, which could result from a different GR conformation. Several studies have appointed this specific phosphorylation as a hallmark for the transactivation potential of the receptor^9, 23, 41^, and consequently, this reduction may explain the observed changes in GRE-driven gene expression. Taken together, our findings suggest an allosteric regulation by CpdA of the DEX-bound receptor.

Combining CpdA with DEX still releases the chaperone protein HSP90, resulting in GR translocation to the nucleus. Phosphorylation of Ser-211 may not be a prerequisite for nuclear translocation, as CpdA does not induce this PTM but still induces (partial) nuclear accumulation of GR^9, 13, 14, 33, 39, 42^. In keeping with this, CpdA/DEX decreased Ser-211 phosphorylation, yet GR nuclear accumulation was unaffected.

Following MARCoNI, we found no significant additional cofactor recruitment by CpdA alone, compared to unliganded GR. In support, Ronacher and colleagues found no recruitment by CpdA-GR of the full-length steroid-receptor coactivator (SRC-1) and GR-interacting protein 1 (GRIP1)^43^. However, other dissociative ligands did support GR coactivator recruitment, albeit with profiles slightly different from the classic agonist DEX. For instance, AL438 showed a similar recruitment of GRIP1 compared to DEX-activated GR, but the interaction with the coactivator PGC-1α was reduced^44^.

Studying the GR cofactor recruitment profile in more detail, we found that the most prominent interaction influenced by CpdA/DEX was between GR and SHP, compared to DEX alone. Borgius and colleagues^45^ showed that SHP inhibits DEX-induced transcriptional activity of the GR in the liver, by antagonizing PGC-1α coactivation of the receptor. One NR-binding LXXLL-related motif, crucial for the inhibitory effect, showed high homology with the NR-binding motif of PGC-1α. As such, this corepressor was able to directly compete with PGC-1α for binding onto the AF-2 domain of GR. Surprisingly, we identified the same LXXLL-related motif within the peptide that interacted significantly more strongly with the DEX/CpdA-activated GR compared to the DEX-bound GR in MARCoNI. This finding suggests that the specific GR profile induced by the ligand combination could be the result of enhanced SHP recruitment following a competition between coactivators. A similar competitive mechanism was proposed for the family member of SHP, DAX-1, that directly modulates GR signalling though binding competition with the coactivator GRIP1. As such, DAX-1 specifically inhibits DEX-induced transactivation, while the GR transrepression potential remains unhampered^46^. Here, the enhanced interaction with CpdA/DEX-stimulated GR was, however, found to be specific for SHP. This finding points to a differential affinity of the two family members for a specific GR conformation, triggered here by CpdA/DEX. Follow-up studies are warranted to further investigate the possible role and underlying mechanisms of SHP, and more generally NR0B family members, on GR-mediated gene regulation and in favouring of GR-mediated transrepression over transactivation.

In conclusion, although CpdA does not stimulate a recombinant GRE-driven reporter on its own, we have shown it can influence the gene regulatory activity of DEX-activated GR. Ligand combinations may induce an advantageous GR-profile in epithelial cells, modulating GR to cooperatively suppress inflammation in combination with enhanced expression of particular anti-inflammatory genes. At the molecular mechanistic level, this unique gene regulatory profile is explained by a decrease in Serine-211 GR phosphorylation coinciding with a stronger recruitment of, among others, the corepressor SHP.

Ligand combinations may be a way forward to obtaining stronger therapeutic effects with lowered concentrations of single compounds, thus possibly circumventing particular side effects.

## Methods

### Cell Culture

Murine L929sA fibrosarcoma cells, human A549 lung epithelial cells and human HEK293T cells were maintained in Dulbecco’s modified Eagle medium (DMEM) supplemented with 10% fetal bovine serum (FBS) and grown at 37 °C under 5% CO_2_. Murine intestinal crypt cells (mIC_cl2_) were a kind gift of the lab of Thomas Brunner^47–49^. These intestinal epithelial cells were maintained in DMEM/Ham’s F-12 (1:1, containing NaHCO3 (2.438 g/L) and L-alanyl-L-glutamine (2 mmol/L, as Glutamax), Gibco) supplemented with 10% charcoal stripped FBS (DCC-FBS), 60 nmol/L sodium selenate, 5 µg/ml apo-transferrin, 10 ng/ml murine EGF, 1 nmol/L triiodothyronine, 5 µg/ml insulin, 20 mmol/L HEPES and Gentamicin. mIC_cl2_ cells were grown at 37 °C under 8% CO_2_.

### Cytokines and Reagents

Recombinant murine TNFα was produced and purified to 99% homogeneity at the VIB protein service facility. A stock solution (1.26 × 10^8^ IU/ml) was prepared in medium, aliquoted and stored at −80 °C. TNFα was used at a final concentration of 2000 IU/ml. Dexamethasone (DEX) was purchased from Sigma-Aldrich. A stock solution was prepared in ethanol (10^−2^ M) and stored in −20 °C. Compound A (CpdA) was synthesized as described by Louw *et al*.^50^. CpdA powder was aliquoted and stored in −80 °C. Before use, the powder was dissolved in ethanol to a stock solution (10^−2^ M). Luciferase (luc) reagent was prepared as previously described^51^.

### Reporter assays

L929sA (by standard CaPO_4_ procedure) and A549 cells (by lentiviral transduction) were stably transfected with p(GRE)_2_-50-Luc+ or p(IL6-κB)_3_-50hu.IL6P-luc+ reporter gene constructs, generated as described previously^51^. Cells were induced as indicated in the figure legends, after which luciferase assays were performed according to the protocol of Promega Corp. For each biological replicate, luciferase measurements were performed at least in triplicate and normalized, where possible, by measurement of β-galactosidase (β-gal) levels with the Galacto-Light kit (Tropix). Light emission was measured with a TopCount NXT luminometer (Perkin-Elmer).

### Cell viability assay

Cell viability assay was performed to quantitate ATP generated by metabolically active cells using a CellTiter-Glo luminescent cell viability assay kit (Promega) according to the manufacturer’s instruction. Briefly, 1.10^5^ cells were cultured in 96-well plates. After 24 h cells were induced with respective compounds as indicated. Subsequently, 25 μl of CellTiter-Glo reagent was added to lyse the cells. After 10 min incubation at room temperature, the luminescence was recorded with the TopCount NXT luminometer (Perkin-Elmer).

### qRT-PCR

A549 or mIC_cl2_ cells were induced as indicated and total solvent concentration was kept similar in all conditions. The cells were serum-starved or steroid-starved with charcoal stripped FBS (DCC-FBS) beforehand as indicated. For the transrepression experiments, A549 cells were serum-starved (see Fig. 2 and Supplementary Figure S2). RNA was isolated using RNeasy Micro Kit (Qiagen) and mRNA was reverse transcribed to cDNA with the PrimeScript RT kit (TaKaRa). cDNA was analysed by real-time PCR with a SYBR Green master mix (Roche). Primer sequences are available on request.

### ELISA

Detection of CCL2 secretion in A549 supernatant was performed according to manufacturer’s protocol using Ready-SET-Go! ELISA Kit (eBioscience).

### SDS-PAGE, Western blot analysis and co-immunoprecipitation

After deprivation of serum, A549 cells were pretreated for 1 h with CpdA (10 µM) and stimulated with DEX (10 nM or 1 µM) for 1 h, 2 h or 5 h. After washing with ice-cold PBS, cell lysates were prepared using SDS sample buffer (62.5 mM Tris-HCl pH 6.8, 2% SDS, 10% glycerol, 0.01% bromophenol blue) followed by standard Western blotting and antibody probing procedures. Equal loading was assayed via a loading control. Anti-GR (H-300) and anti-phospho-Ser-211 GR were purchased from Santa Cruz Biotechnology and Cell Signaling Technology, respectively.

For co-immunoprecipitation, HEK293T cells were transiently transfected using the calcium phosphate precipitation technique with FLAG-SHP and HA-GRα at a ratio of 5 to 1. Twenty-four hours later, cells were serum-starved overnight, 1 h pretreated with CpdA (10 µM) followed by DEX (1 µM) stimulation. Three hours later, cells were lysed (50 mM Tris-HCl pH 7.5, 125 mM NaCl, 5% glycerol, 0.2% NP40, 1.5 mM MgCl_2_ and Complete Protease Inhibitor Cocktail (Roche)) and incubated overnight with anti-FLAG beads (anti-FLAG M2 affinity gel, Sigma Aldrich) on a rotor at 4 °C. These beads were blocked before for 1 h at 4 °C, using undiluted StartingBlock^TM^ (TBS) Blocking buffer (Thermo Scientific). Following overnight incubation and three washing steps, the samples were eluted using Laemmli buffer, boiled for 5 min at 95 °C and stored at −20 °C. Finally, standard western blotting and antibody probing procedures using anti-HA rat (Roche), anti-FLAG rabbit (Sigma Aldrich) and anti-actin (as loading control) mouse antibody (Sigma Aldrich) were performed.

### Immunofluorescence

A549 cells, seeded on coverslips and serum-deprived overnight, were induced as indicated. After fixation, endogenous GRα was visualized using rabbit anti-GR (H-300) antibody (Santa Cruz Biotechnology) followed by donkey Alexa Fluor 488 anti-rabbit IgG (Molecular Probes, Invitrogen). Cell nuclei were stained with DAPI DNA staining (300 nM, Invitrogen).

### MAPPIT

Constructs were generated by standard PCR- or restriction based cloning procedures. The generation of the pCLG-GRα plasmid (GR-bait chimera) and empty prey control was described previously by Beck *et al*.^29^. The prey plasmids pMG2-HSP90AA1 (HSP90-prey chimera), pMG1-DAX-1 and pMG1-SHP were created by Gateway transfer of the full size DAX-1 and SHP ORFs, obtained as entry clones from the hORFeome collection (hORFeome 8.1), into the Gateway compatible pMG1 or pMG2 prey destination vector as described earlier^52^. The pXP2d2-rPAP1-luciferase reporter has been described elsewhere^53^. For the MAPPIT analysis, 10 000 HEK293T cells/well were seeded in 96-well plates. One day later, cells were transiently co-transfected using the calcium phosphate precipitation technique with the GRα-bait plasmid, the STAT3-responsive rPAP1-luc reporter plasmid and the desired prey-plasmid. 24 h after transfection, cells were stimulated with leptin (100 ng/ml) or leptin in combination with DEX (1 µM) and/or CpdA (10 µM) for another 24 h or were left untreated. Luciferase measurements of total cell lysates were performed as described above, and performed in triplicate (normalized by β-galactosidase expression).

### MARCoNI

The MARCoNI assays were performed in a PamStation96 controlled by the EvolveHT software (PamGene International BV). Nuclear Receptor PamChip Arrays (PamGene International BV) with the immobilized coregulatory-derived peptides were incubated with the recombinant protein GRα-LBD-GST (Invitrogen). The reaction mixture for each array consists of 1 nM GRα-LBD-GST and 25 nM Alexa Fluor 488-conjugated anti-GST antibody (Invitrogen) in nuclear receptor buffer F (Invitrogen) in the absence or presence of GR ligands at the indicated concentrations. The MARCoNI procedure has been described in detail previously^25, 26^. Receptor-peptide interaction strengths are reflected by fluorescent signal intensities. Image analysis consists of automated spot finding followed by quantifications using the BioNavigator software (PamGene International BV). The signal-minus-background value was subsequently used as the quantitative parameter for binding. Data was fitted according to a Loess regression using a customized R script.

### Statistical Analysis

A Hierarchical Generalized Linear Mixed Model (HGLMM) (fixed model: poisson distribution, log link; random model: gamma distribution, log link) as implemented in Genstat v18 has been fitted to reporter assay, qPCR, ELISA and MAPPIT data. In case of qPCR, the HGLMM model has been fitted to the data of multiple genes jointly. The linear predictor vector of the fluorescence or luminescence (for ELISA) values can be written as follows:

$$\mathrm{log}(\mu )=\eta ={\bf{X}}\beta +{\bf{Z}}v,$$

where the matrix X is the design matrix for the fixed terms (e.g. for qPCR: GENE, INDUCTION) and their interaction, *β* is their vector of regression coefficients, **Z** is the design matrix for the random term (e.g. REPLICATE), and ν is the corresponding vector of random effects having a gamma distribution. T statistics were used to assess the significance of effects (on the transformed scale) by pairwise comparisons to the reference level (as indicated in the figure legends). Estimated mean values and standard errors (SE’s) were obtained as predictions from the HGLMM, formed on the scale of the response variable.

The MARCoNI data was normalized using Loess regression, followed by ANOVA and Tukey’s multiple comparison post-hoc t-test.

Statistical analyses were carried out using GenStat (Release 18) and GraphPad Prism (version 7) Software.

## Electronic supplementary material

Supplementary Information
