# Supplementary material for: Compound A influences gene regulation of the Dexamethasone-activated glucocorticoid receptor by alternative cofactor recruitment
Source: Sci Rep. 2017 Aug 14;7:8063. doi: 10.1038/s41598-017-07941-y (PMC5556032; doi:10.1038/s41598-017-07941-y)
Supplement: Supplementary file 1 — Supplementary Information [file 41598_2017_7941_MOESM1_ESM.pdf]

Compound A influences gene regulation of the Dexamethasone-activated glucocorticoid receptor by alternative cofactor recruitment

Desmet S.J, Bougarne N, Van Moortel L, De Cauwer L, Thommis J, Vuylsteke M, Ratman D, Houtman R, Tavernier J, De Bosscher K[1,*]

Supplementary Information

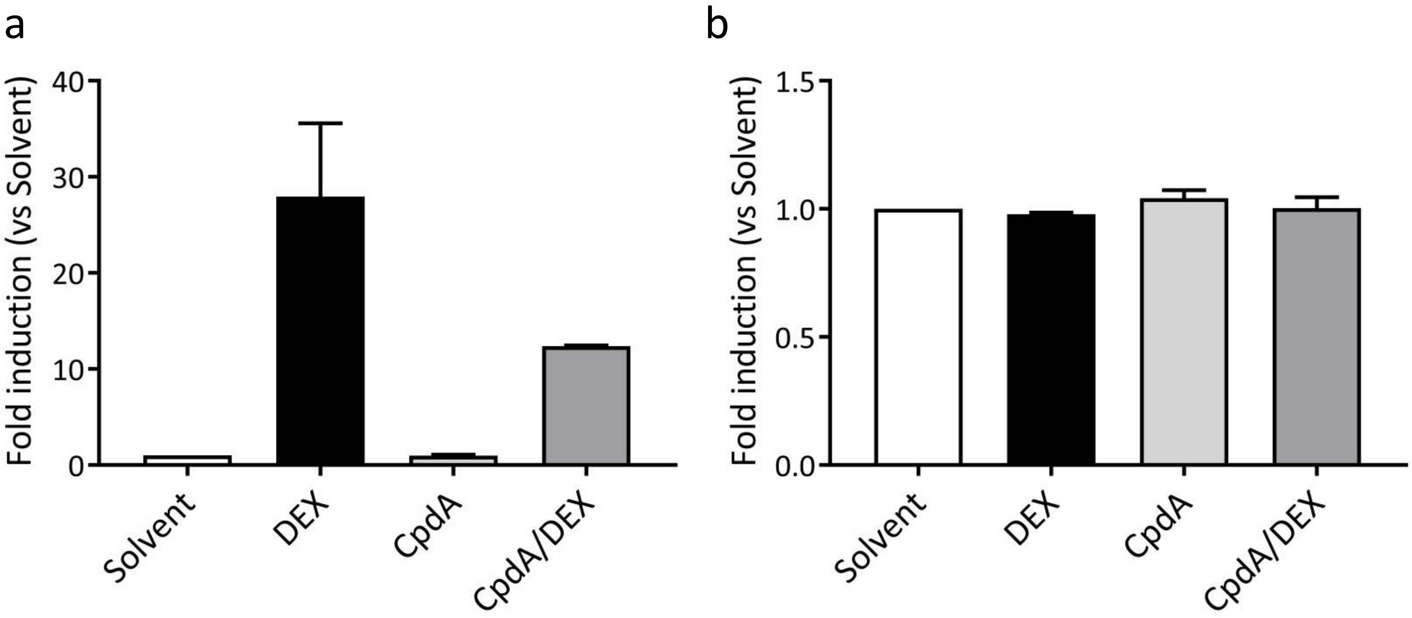

Supplementary figure S1. Combination of DEX with CpdA reduces the transactivation activity on a GRE-dependent reporter.

(**a**) A549 cells with stably integrated $p(GRE)_2$-50-luc+, a GRE-dependent promoter construct, were pre-incubated with CpdA (10µM) for 1h, after which DEX (1µM) was added for 5h. Promoter activities are expressed as relative induction factor versus Solvent (+ SEM, n=2).

(**b**) For the analysis of cell viability, a CellTiter-Glo luminescent cell viability kit was used to measure ATP production. Results are reported as fold change in ATP versus Solvent (+ SEM, n=2).

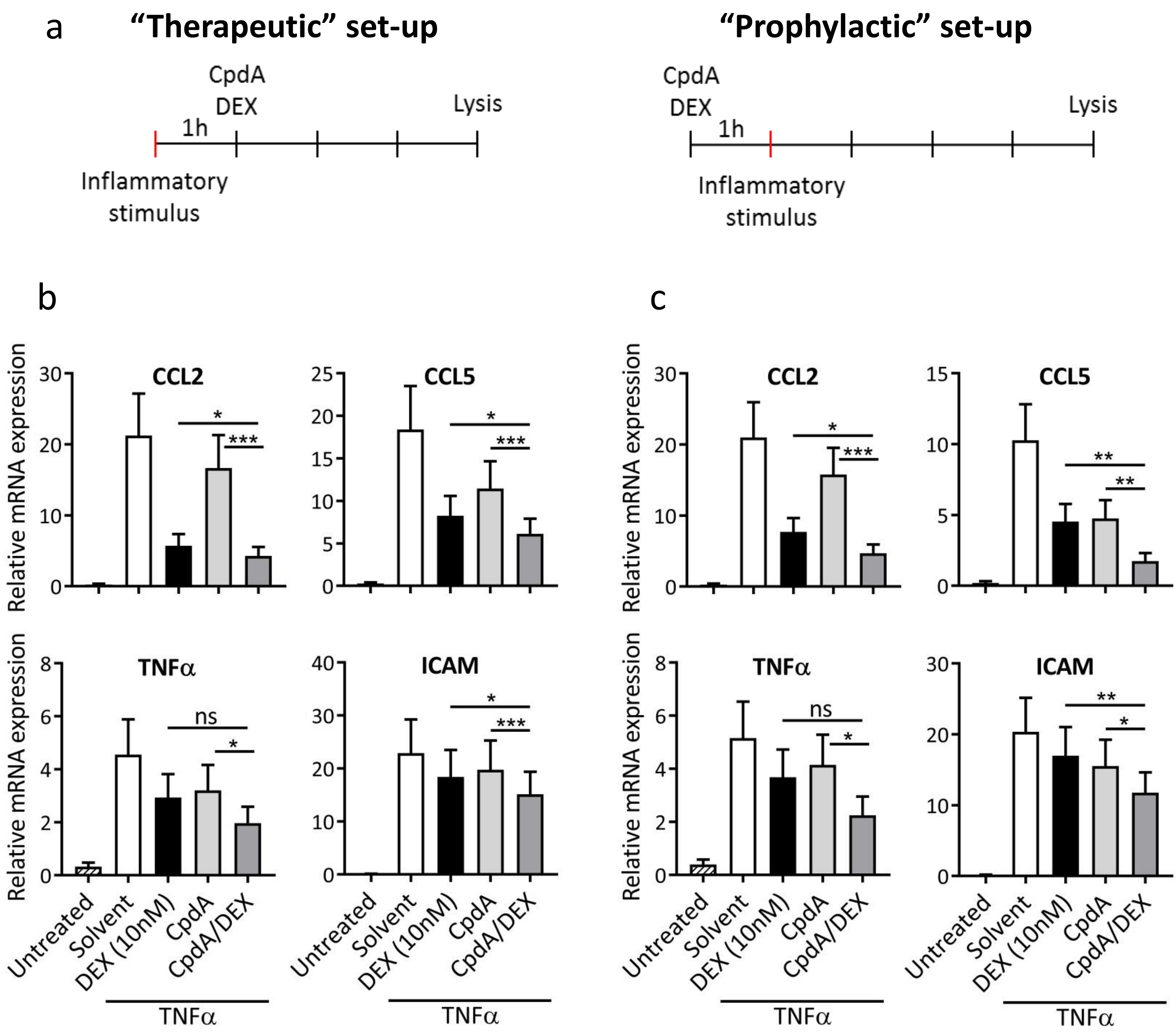

Supplementary figure S2. Simultaneously combining CpdA with a non-saturating DEX concentration suppresses particular inflammatory genes at the mRNA level

(**a**) Scheme of the experimental set-ups

(**b**) A549 cells were stimulated with TNFα (2000 units/ml) for 1h, after which CpdA (10µM) and DEX (10nM) were added for 3h. Total RNA was extracted and subjected to RT-qPCR. Expression values were normalized to the reference genes *b-Actin* and *HPRT* using qBase+*.* Three independent replicates were performed. Means + SE, obtained as predictions from the HGLMM fitted to the data, are shown on the original scale for CCL2, CCL5, TNFα and ICAM.

(**c**) A549 cells were stimulated with CpdA (10µM) and DEX (10nM) for 1h, after which TNFα (2000 units/ml) was added for 4h. Total RNA was extracted and subjected to RT-qPCR. Expression values were normalized to the reference genes *b-Actin* and *HPRT* using qBase+*.* Three independent replicates were performed. Means + SE, obtained as predictions from the HGLMM fitted to the data, are shown on the original scale for CCL2, CCL5, TNFα and ICAM.

The significance of gene-specific ligand combination effects on inflammatory gene expression, estimated as differences (on the transformed scale) to the gene-specific reference level CpdA/DEX, were assessed using a t-test (*: $p<0.05$; **: $p<0.01$; ***: $p<0.001$).

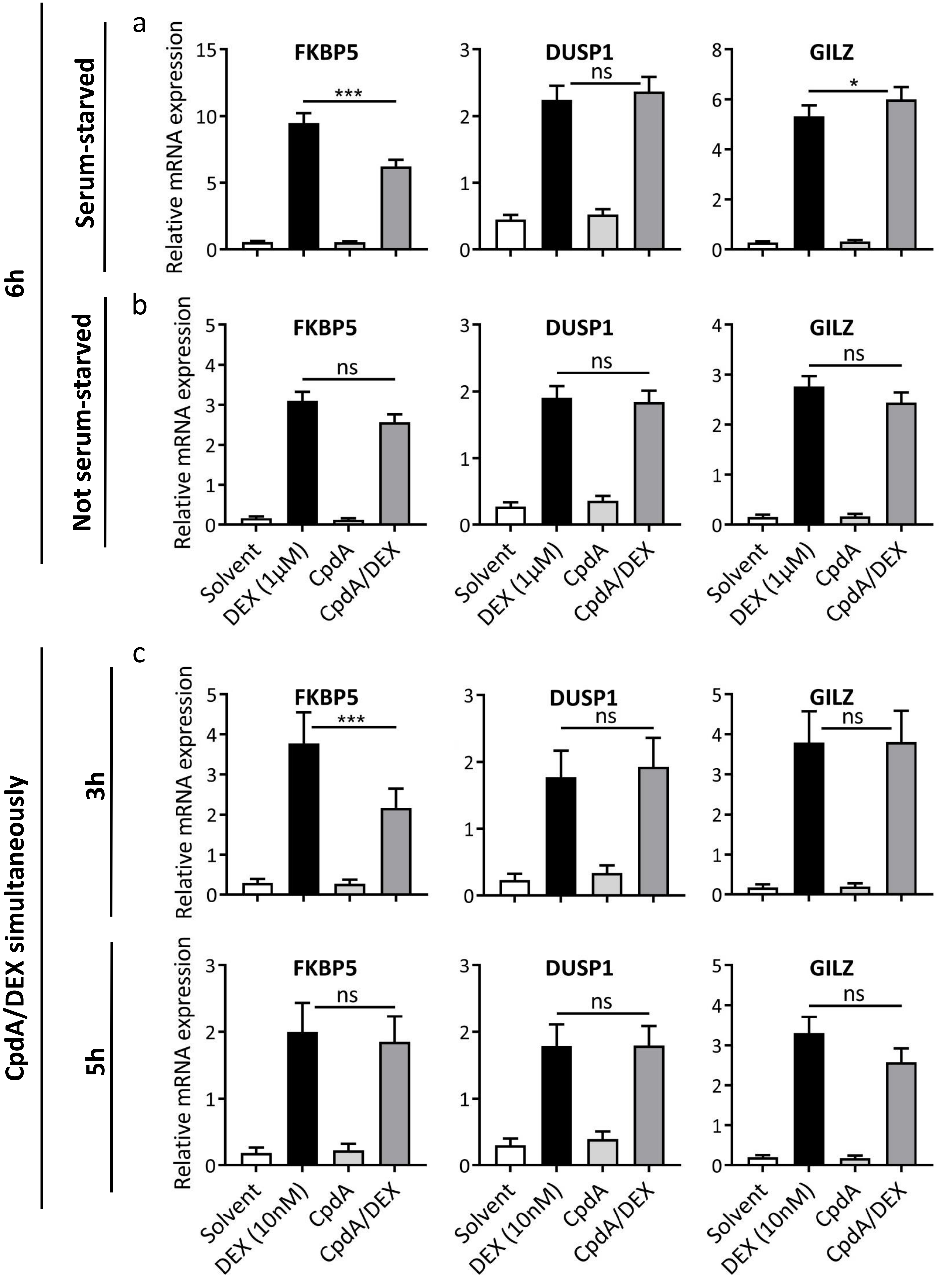
6h
Serum-starved
Not serum-starved
CpdA/DEX simultaneously
3h
5h
a
b
c
FKBP5
DUSP1
GILZ
Relative mRNA expression
***
ns
*
Solvent
DEX (1µM)
DEX (10nM)
CpdA
CpdA/DEX

Supplementary figure S3. The effect of CpdA stimulation on DEX-induced gene expression is gene-specific
(**a**) Serum-starved A549 cells were pre-incubated with CpdA (10µM) for 1h, after which DEX (1µM) was added for 5h. Total RNA was extracted and subjected to RT-qPCR. Expression values were normalized to the reference genes *Cyclo* and *HPRT* using qBase+. Five independent replicates were performed. Means + SE, obtained as predictions from the HGLMM fitted to the data, are shown on the original scale for FKBP5, GILZ and DUSP1.
(**b**) A549 cells were pre-incubated with CpdA (10µM) for 1h, after which DEX (1µM) was added for 5h. Total RNA was extracted and subjected to RT-qPCR. Expression values were normalized to the reference genes *B2M* and *HPRT* using qBase+. Three independent replicates were performed. Means + SE, obtained as predictions from the HGLMM fitted to the data, are shown on the original scale for FKBP5, DUSP1 and GILZ
(**c**) A549 cells were simultaneously stimulated with CpdA (10µM) and DEX (10nM) for 3h or 5h. Total RNA was extracted and subjected to RT-qPCR. Expression values were normalized to the reference genes *b-Actin* and *HPRT* using qBase+. Three independent replicates were performed. Means + SE, obtained as predictions from the HGLMM fitted to the data, are shown on the original scale for FKBP5, DUSP1 and GILZ.

The significance of gene-specific CpdA effects on DEX-induced gene expression, estimated as differences (on the transformed scale) to the gene-specific reference level CpdA/DEX, were assessed using a t-test (*: $p<0.05$; **: $p<0.01$; ***: $p<0.001$).

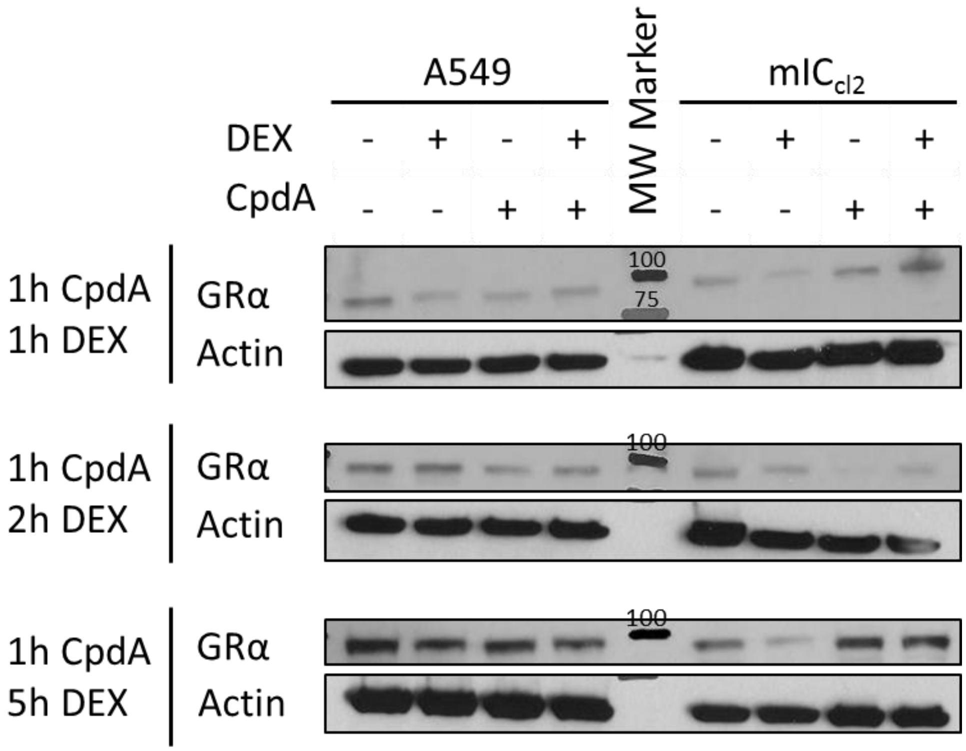

Supplementary figure S4. The GR protein levels in A549 and mIC_{cl2} cells

A549 and mIC_{cl2} cells were pre-incubated with CpdA (10µM) for 1h, after which DEX (10nM) was added for 1h, 2h or 5h. Total cell lysates were prepared and subjected to Western Blot analysis. Actin served as a loading control. The middle lane was used to mark the molecular weight standard (n=2, representative figure).
